# Enhanced Replication of Highly Pathogenic Influenza A(H7N9) Virus in Humans 

**DOI:** 10.3201/eid2404.171509

**Published:** 2018-04

**Authors:** Seiya Yamayoshi, Maki Kiso, Atsuhiro Yasuhara, Mutsumi Ito, Yuelong Shu, Yoshihiro Kawaoka

**Affiliations:** University of Tokyo, Tokyo, Japan (S. Yamayoshi, M. Kiso, A. Yasuhara, M. Ito, Y. Kawaoka);; Sun Yat-Sen University, Shenzhen, China (Y. Shu);; Chinese Centers for Disease Control and Prevention, Beijing, China (Y. Shu);; University of Wisconsin–Madison, Madison, Wisconsin, USA, and Japan Science and Technology Agency, Saitama, Japan (Y. Kawaoka)

**Keywords:** Highly pathogenic, H7N9, PB2, PA, enhanced polymerase activity, influenza, viruses, respiratory infections

## Abstract

To clarify the threat posed by emergence of highly pathogenic influenza A(H7N9) virus infection among humans, we characterized the viral polymerase complex. Polymerase basic 2–482R, polymerase basic 2–588V, and polymerase acidic–497R individually or additively enhanced virus polymerase activity, indicating that multiple replication-enhancing mutations in 1 isolate may contribute to virulence.

Highly pathogenic influenza A(H7N9) virus has infected humans every influenza season since 2013; the fifth epidemic wave occurred during the 2016‒17 season (*1*,*2*). Since 2013, a total of 1,565 laboratory-confirmed human cases and 612 related deaths have been reported (http://www.who.int/influenza/human_animal_interface/Influenza_Summary_IRA_HA_interface_12_07_2017.pdf?ua = 1). During the fifth wave, H7N9 viruses possessing hemagglutinin with multibasic amino acids at the cleavage site were isolated from birds and humans (*2*–*4*). H7N9 isolates from humans possessed hemagglutinin with a preference for human-type receptors and neuraminidase with inhibitor resistance (*4*,*5*); one of those isolates transmitted among ferrets via respiratory droplets (*6*). Emergence of highly pathogenic H7N9 viruses with such properties is a serious threat to public health. Full comprehension of the extent of this threat requires detailed characterization of these viruses.

## The Study

We attempted to identify the replication-enhancing amino acids in the polymerase complex of H7N9 virus A/Guangdong/17SF003/2016 (GD), which was isolated from the first reported H7N9-infected patient (*3*,*5*) and harbors polymerase basic (PB) 2 with 271T, 588V, 591Q, 627E, and 701D. Amino acids at these positions are known to alter viral polymerase activity in mammalian and avian cells at different temperatures (*7*–*11*).

We compared the viral polymerase activity of wild-type GD with that of A/Anhui/1/2013(H7N9) virus (AN) in human A549 cells at 33°C or 37°C (temperatures of the human upper and lower respiratory tract) and in chicken DF-1 cells at 39°C (body temperature of birds). Although both viruses exhibited comparable activity in DF-1 cells, AN activity was higher than GD activity in A549 cells at both temperatures because wild-type AN/PB2 acquired polymerase activity–enhancing K at position 627 of PB2 during replication in the infected human (*8*). We therefore tested AN/PB2-627E, which possesses an avian ancestral amino acid in PB2-627, and AN/PB2-627E-701N, which possesses polymerase-enhancing PB2-701N (*8*). In human A549 cells, wild-type GD showed viral polymerase activity comparable to that of AN/PB2-627E-701N Technical Appendix Figure 1, panel A). These results indicate that the viral polymerase activity of wild-type GD in mammalian cells has increased more than that of virus bearing avian-like ancestral AN/PB2–627E. 

To determine which component of the viral replication complex (PB2, PB1, polymerase acidic [PA], or nucleoprotein) contributes to the activity of the GD polymerase complex, we tested the polymerase activity of GD replication complexes in which we had replaced each viral protein with its AN/PB2-627E counterpart. We found that the viral polymerase activity in A549 cells was remarkably decreased by AN/PB2-627E and moderately decreased by AN-PA (Technical Appendix Figure 1, panel B). These results suggest that the PB2 and the PA of GD are involved in the relatively high polymerase activity of the GD replication complex.

**Figure 1:**
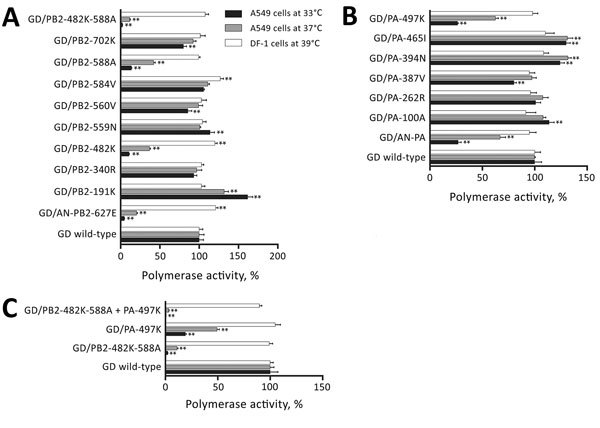
Viral polymerase activity of wild-type, PB2 mutant, and PA mutant polymerase complexes. A) Viral polymerase activities of highly pathogenic influenza A(H7N9) virus GD replication complexes harboring amino acid substitutions in PB2 (A), PA (B), or PB2 and PA (C) in human A549 and chicken DF-1 cells. The data shown are relative polymerase activities ± SD (n = 3). The polymerase activity of GD wild-type was set to 100%. **p<0.01, according to a 1-way analysis of variance followed by a Dunnett test. Error bars indicate SD. AN, A/Anhui/1/2013(H7N9); GD, A/Guangdong/17SF003/2016; PA, polymerase acidic; PB, polymerase basic.

When we compared the amino acid sequences of GD-PB2 and GD-PA with those of AN/PB2-627E and AN-PA, we found 8 and 6 differences, respectively (Table 1). To identify which substitutions contributed to the enhanced polymerase activity, we constructed a series of plasmids encoding GD-PB2 or GD-PA harboring single substitutions and examined polymerase activity. Of the 8 PB2 mutants, GD/PB2-482K and GD/PB2-588A drastically reduced viral polymerase activity in A549 cells, although this activity was slightly higher than that of AN/PB2-627E (Figure 1, panel A). Therefore, we tested the viral polymerase activity of GD-PB2 possessing both mutations (GD/PB2-482K-588A) and found a further decrease in the double mutant. Of the 6 PA mutants, GD/PA-497K showed reduced viral polymerase activity in A549 cells (Figure 1, panel B). Compared with GD/PB2-482K-588A or GD/PA-497K alone, the polymerase activity of GD/PB2-482K-588A plus GD/PA-497K was further reduced (Figure 1, panel C). Collectively, these data demonstrate that PB-482R, PB2-588V, and PA-497R play crucial roles in the enhanced activity of the GD polymerase complex.

**Table 1:** Amino acid differences between PB2 and PA in 2 influenza A(H7N9) viruses*

Virus	PB2									PA					
191	340	482	559	560	584	588	702	100	262	387	394	465	497	
GD wild-type	E	K	R	T	I	I	V	R		V	K	I	D	V	R	
AN/PB2-627E†	K	R	K	N	V	V	A	K		A	R	V	N	I	K	

*AN, A/Anhui/1/2013(H7N9); GD, A/Guangdong/17SF003/2016; PA, polymerase acidic; PB, polymerase basic.  †This mutant possessed a K to E substitution at position 627 of PB2, but the other residues of PB2 and PA were identical to those of wild-type AN.

To examine the role of these amino acids on virus growth in human cells, we prepared wild-type and mutant viruses in the GD background by using reverse genetics. GD/PB2-482K and GD/PB2-588A viruses replicated less efficiently than wild-type GD virus in A549 cells (Technical Appendix Figure 2, panels A, B). Replication of GD/PB2-482K-588A and GD/PB2-482K-588A+PA-497K viruses was less efficient than that of GD/PB2-482K and GD/PB2-588A viruses. GD/PA-497K virus showed growth comparable to that of the wild-type GD virus. In DF-1 cells, all tested viruses produced similar growth curves (Technical Appendix Figure 2, panel C). These results indicate that PB2-482R and PB2-588V play a central role in enhancing virus replication in mammalian cells.

To assess the role of these amino acids in vivo, we compared virus titers in the lungs, nasal turbinates, and brains of mice infected intranasally with 10^2^ PFU of each virus. On day 3 after infection, we did not detect GD/PB2-482K, GD/PB2-482K-588A, or GD/PB2-482K-588A+PA-497K viruses in the lungs or turbinates (Table 2). Replication of GD/PB2-588A or GD/PA-497K virus was significantly decreased in the lungs and slightly decreased in the turbinates. On day 6 after infection, we found similar trends to those observed on day 3. Wild-type GD virus was detected in the brain of 1 of 3 mice. 

**Table 2:** Titers of influenza A(H7N9) GD virus in organs of experimentally infected mice*

Virus	Postinfection day 3, mean log_10_ PFU ± SD/g				Postinfection day 6, mean log_10_ PFU ± SD/g		
Lung	NT	Brain	Lung	NT	Brain
GD wild-type	5.7 ± 0.2	4.2 ± 0.8	ND		5.8 ± 0.4	5.9 ± 0.7	3.2
GD/PB2-482K	ND, p<0.01	ND, p<0.01	ND		3.0, 3.4, p<0.01	2.6 ± 0.4, p<0.05	ND
GD/PB2-588A	3.3 ± 0.6, p<0.01	3.1, p<0.01	ND		4.6 ± 0.8	4.4 ± 2.5	ND
GD/PB2-482K-588A	ND, p<0.01	ND, p<0.01	ND		3.1 ± 0.3, p<0.05	ND, p<0.01	ND
GD/PA-497K	5.1 ± 0.2, p<0.05	2.9 ± 1.0	ND		4.6 ± 1.0	5.8 ± 0.4	ND
GD/PB2-482K-588A+PA-497K	ND, p<0.01	ND, p<0.01	ND		2.2, 2.7, p<0.01	ND, p<0.01	ND

*BALB/c mice were intranasally inoculated with 10^2^ PFU of virus (in 50 μL). Three animals per group were euthanized on postinfection days 3 and 6. Statistically significant differences compared with GD wild-type–infected mice were determined by use of a 1-way analysis of variance followed by a Dunnett test. GD, A/Guangdong/17SF003/2016; ND, virus not detected (detection limit ≈2 log_10_ PFU/g); NT, nasal turbinates; PA, polymerase acidic; PB, polymerase basic.

**Figure 2:**
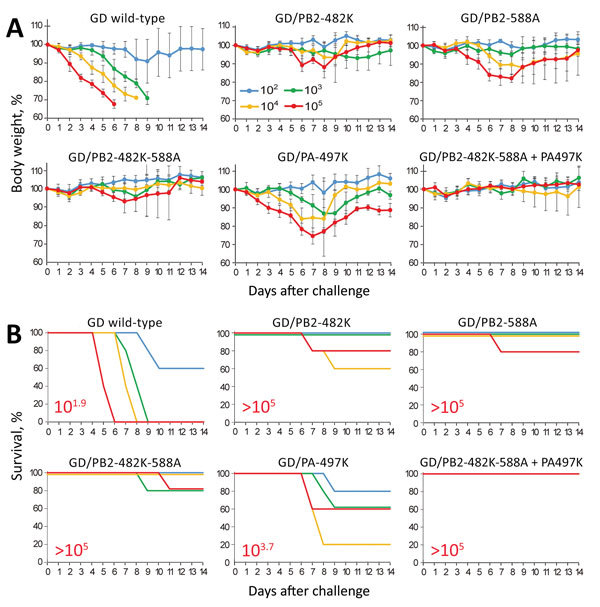
Virulence of wild-type and mutant highly pathogenic influenza A(H7N9) virus GD viruses in mice. Five mice per group were intranasally inoculated with 10^2^, 10^3^, 10^4^, or 10^5^ PFU (each in 50 μL) of the indicated viruses. Body weight (A) and survival (B) were monitored daily for 14 days. A) The values represent the average body weight ± SD compared with the baseline weight from 5 mice. Two-way analysis of variance followed by a Dunnett test revealed that the body weight loss of mice infected with each mutant virus at any dose was significantly reduced compared with that of mice infected with GD wild-type virus (p<0.01). B) The 50% lethal doses for mice (in red) were calculated according to the Spearman-Karber method. Error bars indicate SD. GD, A/Guangdong/17SF003/2016; PA, polymerase acidic; PB, polymerase basic.

Next, we evaluated virus pathogenicity in mice infected with 10^2^‒10^5^ PFU of each virus by monitoring changes in body weight. When inoculated with wild-type GD virus, almost all mice had to be euthanized, resulting in a 50% mouse lethal dose (MLD_50_) of 10^1.9^ PFU (Figure 2, panels A and B). Transient or severe weight loss was caused by GD/PB2-482K, GD/PB2-588A, and GD/PB2-482K-588A in 1‒3 of 20 euthanized mice and by GD/PA-497K viruses in 9 of 20 euthanized mice; MLD_50_ values were higher than these for wild-type GD virus. The GD/PB2-482K-588A+PA-497K virus did not affect body weight (MLD_50_ >10^5^ PFU). Virulence of GD/PB2-588A in mice was comparable to that of GD/PB2-482K and GD/PB2-482K-588A, although GD/PB2-588A replicated better in the lungs and turbinates than GD/PB2-482K and GD/PB2-482K-588A (Table 2); however, the levels of GD/PB2-588A replication in mice were lower than those of wild-type GD, resulting in reduced pathogenicity in mice. These results demonstrate that PB2-482R and PB2-588V contribute to high virulence in mice and that PA-497R is also involved.

## Conclusions

We demonstrated that PB2-482R, PB2-588V, and PA-497R contribute to the enhanced polymerase activity of highly pathogenic H7N9 virus. These mutations additively increase viral polymerase activity and pathogenicity. PB2-482R is present in 0.79% (7/884) of human-derived H7N9 viruses, PB2-588V in 16.6% (147/883), and PA-497R in 0.81% (7/862) (Technical Appendix Table). Of 31 highly pathogenic H7N9 viruses isolated from humans, 5 (16.1%) possessed PB2-482R and PA-497R, 12 (38.7%) PB2-627K, and 10 (32.3%) PB2-588V (Technical Appendix Figure 3, panel A). Although conventional replication-enhancing amino acids (PB2-591R, PB2-627K, and PB2-701N) rarely coexist in 1 PB2 molecule, PB2-588V clearly permits acquisition of additional mutations, such as PB2-482R and PB2-627K (*12*). These double substitutions could have an additive effect on virulence enhancement, as also shown previously (*9*), suggesting that in mammalian hosts, these double-mutant viruses may be fitter than single-mutant viruses. Therefore, future H7N9 virus surveillance studies should take into consideration single markers and combinations of markers.

PB2-482R was located in 1 of 2 nuclear localization signals spanning amino acids 449 to 495 (*13*) within the cap-binding domain of the influenza A virus polymerase complex (Technical Appendix Figure 3, panel B). PB2-588V was located near PB2-627K in the 627 domain. PA-497R was located at 1 of 2 binding sites for transcriptional activator hCLE (*14*) but was not exposed on the protein surface. PB2-588V is probably involved in ANP32A-dependent high polymerase activity in mammalian hosts (*15*), and the role of PB2-482R might differ from that of other polymerase-enhancing amino acids in PB2.

Technical Appendix Additional methods and details used in study of enhanced replication of a highly pathogenic influenza A(H7N9) virus in humans.
