# Supplementary material for: Enhanced Replication of Highly Pathogenic Influenza A(H7N9) Virus in Humans
Source: Emerg Infect Dis. 2018 Apr;24(4):746–50. doi: 10.3201/eid2404.171509 (PMC5875272; doi:10.3201/eid2404.171509)
Supplement: Technical Appendix — Additional methods and details used in study of enhanced replication of a highly pathogenic influenza A(H7N9) virus in humans. [file 17-1509-Techapp-s1.pdf]

Article DOI: https://doi.org/10.3201/eid2404.171509

Enhanced Replication of Highly Pathogenic Influenza A(H7N9) Virus in Humans

Technical Appendix

Additional Methods and Details

Ethics and biosafety statements. All experiments with H5N1 and H7N9 viruses were performed in biosafety level 3 (BSL3) laboratories at the University of Tokyo, which were approved for such use by the Ministry of Agriculture, Forestry, and Fisheries, Japan. All experiments with mice were performed in accordance with the University of Tokyo's Regulations for Animal Care and Use and were approved by the Animal Experiment Committee of the Institute of Medical Science, the University of Tokyo.

Cells. Madin-Darby canine kidney (MDCK) cells were maintained in Eagle's minimal essential medium (MEM) containing 5% newborn calf serum (NCS). Human embryonic kidney 293T cells and chicken fibroblast DF-1 cells were maintained in Dulbecco's modified Eagle's medium (DMEM) containing 10% fetal calf serum (FCS). Human alveolar adenocarcinoma epithelial A549 cells were maintained in Ham's F12-K containing 10% FCS. MDCK, 293T, and A549 cells were maintained at 37°C under 5% CO_2. DF-1 cells were maintained at 39°C under 5% CO_2.

Plasmids. Viral RNA expression plasmids (pHH21) encoding each of the eight virus segments and protein expression plasmids (pCAGGS) encoding PB2, PB1, PA, and NP derived from A/Anhui/1/2013 (H7N9; AN) or A/Guangdong/17SF003/2016 (H7N9; GD) were

previously described (*1*). Plasmids encoding PB2 mutants of A/Anhui/1/2013 (AN/PB2–627E and AN/PB2–627E-701N) were also described previously (*1*).

Mutations in the PB2 and PA genes were generated by PCR amplification of the RNA polymerase I plasmid for the PB2 and PA segments with primers possessing the desired mutations (primer sequences available upon request). The PCR products were cloned into pHH21 or pCAGGS/MCS. All constructs were sequenced to confirm the absence of unwanted mutations.

Minigenome assay. A minigenome assay based on the dual-luciferase system was performed as previously reported (*2*,*3*). Briefly, human A549 and chicken DF-1 cells were transfected with viral protein expression plasmids for NP, PB1, PB2 or its mutants, and PA or its mutants (0.2 µg each), with a plasmid expressing a reporter vRNA encoding the firefly luciferase gene under the control of the human or chicken RNA polymerase I promoter (pPolI/NP(0)Fluc(0) or pPolIGG-NP(0)Fluc(0), respectively, 0.2 µg each), and pRL null (Promega, 0.2 µg), which expresses Renilla luciferase, as a transfection control. The luciferase activities in the A549 cells incubated at 33 or 37°C and the DF-1 cells incubated at 39°C were measured by using the Dual-Glo Luciferase Assay System (Promega) at 24 h post-transfection. Polymerase activity was calculated by standardization of the firefly luciferase activity to the Renilla luciferase activity. Relative polymerase activities are presented in the figures.

Reverse genetics. Plasmid-based reverse genetics for virus generation was performed as previously described (*4*). Briefly, eight RNA polymerase I plasmids (for the synthesis of the eight influenza A viral RNAs) together with plasmids for the expression of the PB2, PB1, PA, and NP proteins derived from the influenza A virus strain A/WSN/33 (H1N1) were transfected into 293T cells. At 24 h post-transfection, culture supernatants were harvested and inoculated

into embryonated chicken eggs for virus propagation. After 1 or 2 days, allantoic fluids containing viruses were collected and centrifuged to remove cell debris and the supernatants were stored as stock viruses. The titers of the stock viruses were determined by means of plaque assays in MDCK cells. All viruses were sequenced to confirm the absence of unwanted mutations.

Growth kinetics of viruses in cell culture. The growth kinetics of the viruses was assessed as previously described (*1*). Briefly, A549 and DF-1 cells were infected with the indicated viruses at a multiplicity of infection (MOI) of 0.001. After incubation at 37°C for 1 h, the viral inoculum was replaced with MEM containing 0.3% bovine serum albumin and TPCK-treated trypsin (0.3 µg/ml), followed by further incubation at 33 or 37°C for A549 cells or 39°C for DF-1 cells. Cell culture supernatants were collected at 24, 48, and 72 h post-infection and subjected to virus titration by use of plaque assays in MDCK cells.

Experimental infection of mice. Baseline bodyweights of all mice were measured before infection. Five 6-week-old female BALB/c mice (Japan SLC) per group were intranasally inoculated with 10^2–10^5 PFUs (PFU) (in 50 µl) of the indicated viruses under anesthesia. Bodyweight and survival were monitored daily for 14 days; mice with bodyweight loss of more than 25% of their baseline bodyweight were euthanized. For virologic examinations, six mice per group were intranasally infected with 10^2 PFU (in 50 µl) of the viruses and three mice per group were euthanized at 3 and 6 days post-infection. The virus titers in the nasal turbinate, lung, and brain were determined by means of plaque assays in MDCK cells.

Phylogenetic analysis. The phylogenetic tree of the HA nucleotide sequences derived from the H7N9 viruses was constructed by using the neighbor-joining (NJ) method with the Kimura distances and the bootstrap procedure (n = 1000) using ClustalW 2.1 on the DDBJ

(DNA Data Bank of Japan) Web site (http://clustalw.ddbj.nig.ac.jp/) and was visualized by using the MEGA 7.0 software. Sequence data for HA, PB2, and PA were obtained from GISAID databases on August 31, 2017. The sequencing dataset used in this study is available upon request.

Molecular modeling. The structural model of the polymerase complex derived from A/little yellow-shouldered bat/Guatemala/060/2010 (H17N10) (PDB code, 4WSB) was used to assign the amino acid positions in the influenza A virus polymerase complex with the PyMOL Molecular Graphics System, version 1.3.

References

1. Yamayoshi S, Fukuyama S, Yamada S, Zhao D, Murakami S, Uraki R, et al. Amino acids substitutions in the PB2 protein of H7N9 influenza A viruses are important for virulence in mammalian hosts. Sci Rep. 2015;5:8039. PubMed http://dx.doi.org/10.1038/srep08039

2. Yamayoshi S, Yamada S, Fukuyama S, Murakami S, Zhao D, Uraki R, et al. Virulence-affecting amino acid changes in the PA protein of H7N9 influenza A viruses. J Virol. 2014;88:3127–34. PubMed http://dx.doi.org/10.1128/JVI.03155-13

3. Imai M, Watanabe T, Kiso M, Nakajima N, Yamayoshi S, Iwatsuki-Horimoto K, et al. A Highly Pathogenic Avian H7N9 Influenza Virus Isolated from A Human Is Lethal in Some Ferrets Infected via Respiratory Droplets. Cell host & microbe. 2017 Nov 8;22(5):615–26 e8.

4. Neumann G, Watanabe T, Ito H, Watanabe S, Goto H, Gao P, et al. Generation of influenza A viruses entirely from cloned cDNAs. Proc Natl Acad Sci U S A. 1999;96:9345–50. PubMed http://dx.doi.org/10.1073/pnas.96.16.9345

Technical Appendix Table. Percentage of isolates with the indicated amino acid residue at each position*

		PB2						PA	
		482		588				497	
Subtype	Host	K	**R†**	A	T	I	**V†**	K	**R†**
Pre2009 H1N1	Human	99.9	0.11	0.89	0.33	97.8	0.9	99.9	0
H1N1pdm09	Human	99.8	0.2	0.08	97.3	0.26	0	99.9	0.05
H3N2	Human	99.9	0.04	0.04	29.5	68.5	1.8	98.7	1.29
H5N1	Human	100	0	93.7	5.93	0.37	0	94.1	5.92
H7N9	Human	99.2	0.79	82.9	0.34	0.11	16.6	98.2	0.81
H1N1	Swine	99.1	0.84	38.0	55.6	3.66	0.92	94.4	5.5
H1N2	Swine	98.6	1.45	19.2	74.7	4.92	0.72	79.3	20.5
H3N2	Swine	98.6	1.2	6.73	85.1	6.73	0.98	68.7	31.4
H5N1	Avian	100	0	93.7	5.93	0.37	0	94.1	5.9
H7N9	Avian	100	0	93.3	0	0.32	6.39	100	0
Other (i.e., not H5N1 or H7N9	Avian	99.5	0.43	92.3	3.26	0.59	3.72	99.3	0.66

*The percentages are based on inspection of ≈900, 3830, 7590, 270, 870, 1310, 690, 920, 270, 310, and 6780 sequences of PB2 or PA derived from human pre2009 H1N1, H1N1pdm09, H3N2, H5N1, H7N9, swine H1N1, H1N2, H3N2, avian H5N1, H7N9, and other virus subtypes, respectively.

†Residues involved in enhanced replication are shown in boldface.

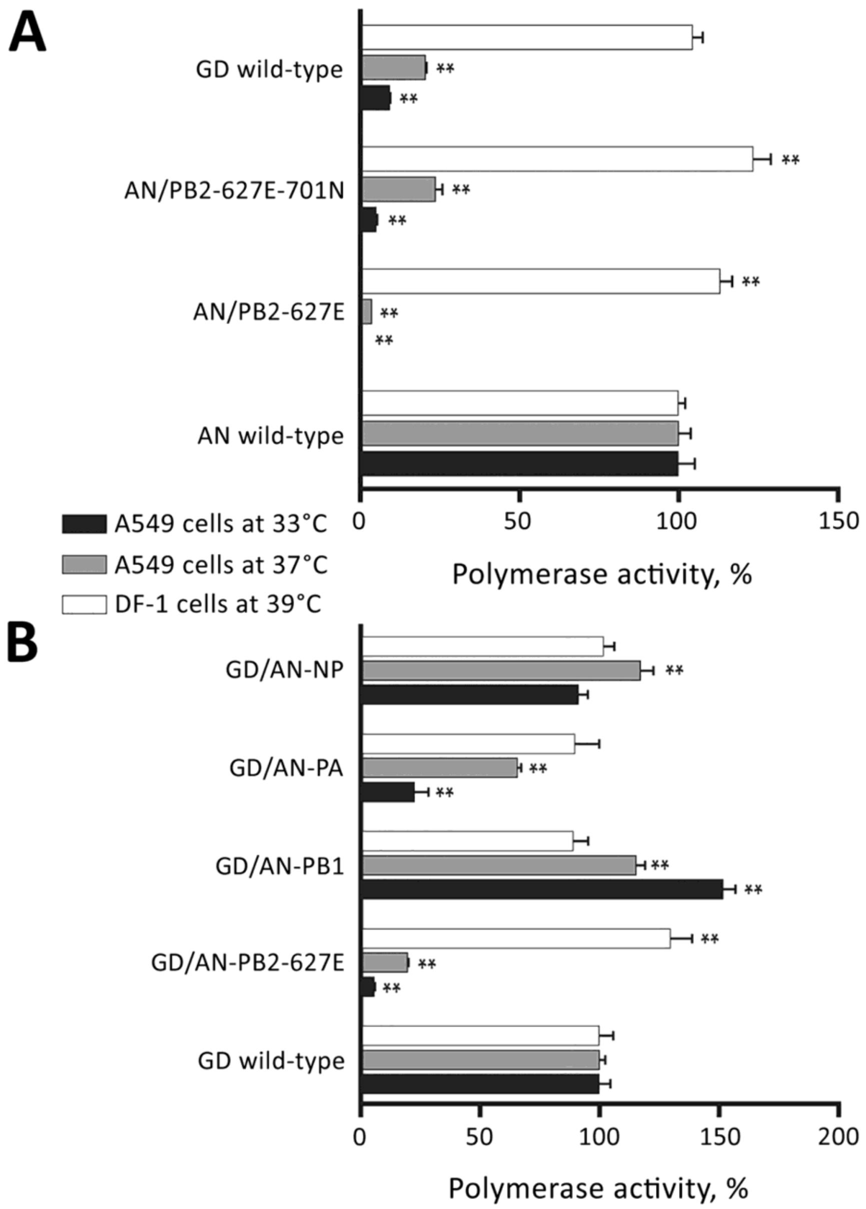

Technical Appendix Figure 1. Each GD-PB2 and -PA is required for enhanced viral polymerase activity in human cells. Viral polymerase activity of wild-type AN, AN-PB2 mutants, and wild-type GD (A) or wild-type and reassortant GD (B) polymerase complex in human A549 and avian DF-1 cells. The viral polymerase activity AN wild-type (A) or GD wild-type (B) was set to 100%. The data shown are relative viral polymerase activities±SD (n = 3). **p < 0.01, according to a one-way ANOVA followed by a Dunnett's test.

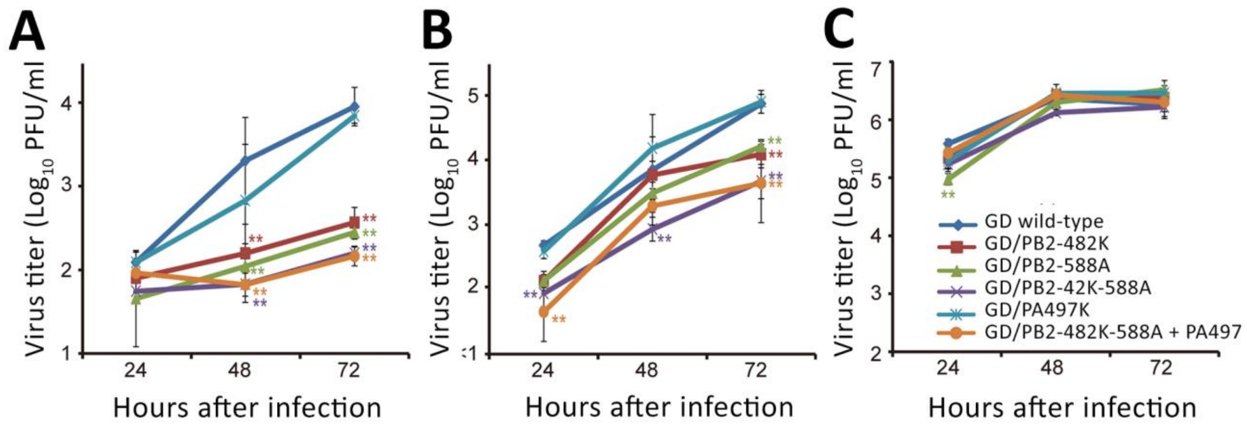

Technical Appendix Figure 2. Growth kinetics of wild-type and mutant GD viruses in human and avian cells. Human A549 cells and chicken DF-1 cells were infected with the wild-type or indicated mutant viruses at an MOI of 0.001 and incubated at 33°C (A) or 37°C (B) for A549 cells and 39°C for DF-1 cells (C). The culture media from the infected cells was collected at the indicated time points. Virus titers were determined by use of a plaque assay in MDCK cells. The average virus titers±SD are plotted (n = 3). $**p < 0.01$, according to a two-way analysis of variance (ANOVA) followed by a Dunnett’s test.

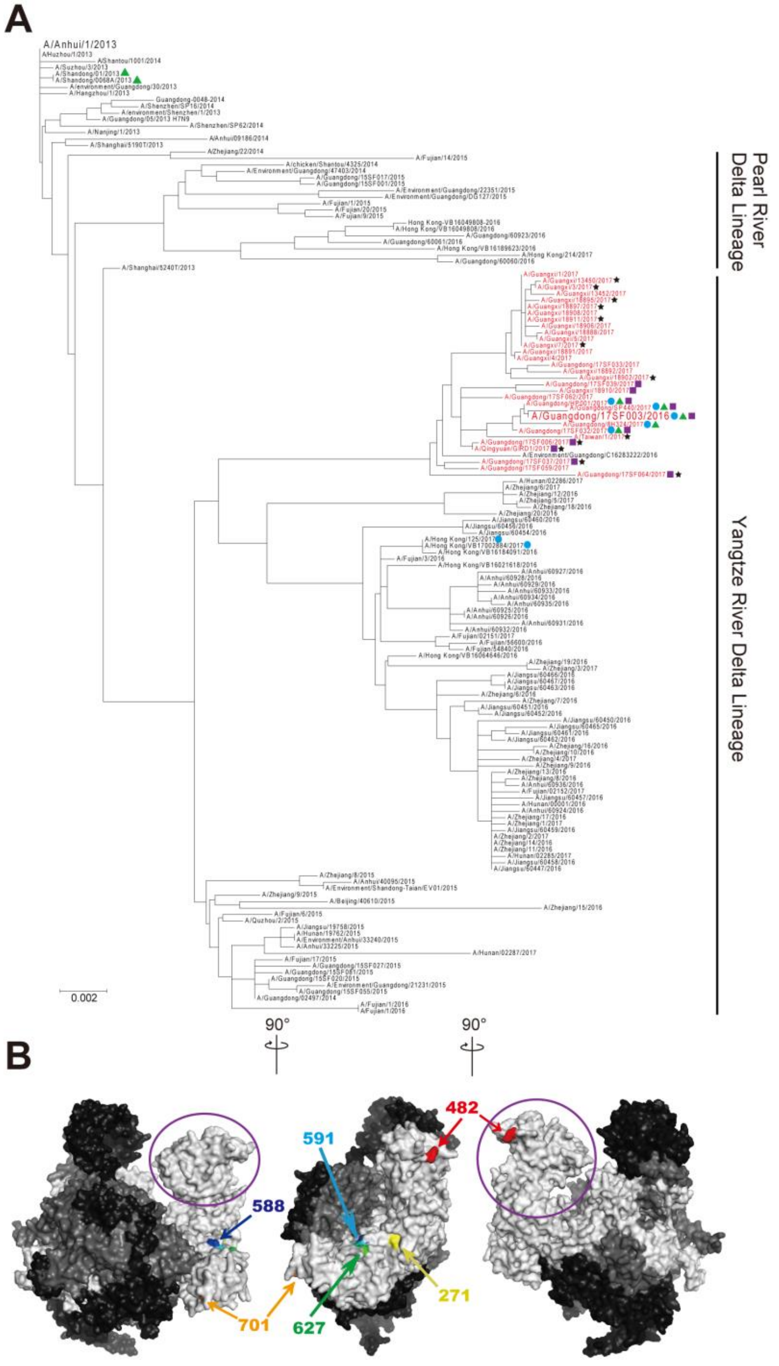

Technical Appendix Figure 3. Phylogenic and structural position of PB2–482R and PA-497R. (A) Phylogenetic tree based on H7N9 HA sequences. Virus isolates marked by cyan circles or green triangles possess PB2–482R or PA-497R, respectively. Highly pathogenic isolates marked by purple squares or black stars possess PB2–588V or PB2–627K, respectively. Red letters indicate highly pathogenic

isolates. (B) Localization of the mammalian adaptive amino acids in the polymerase complex. The polymerase subunits PB2, PB1, and PA are shown as white, gray, and black, respectively. Each amino acid (PB2–271A, PB2–482R, PB2–588V, PB2–591K, PB2–627K, and PB2–701N) of PB2 that is involved in enhanced replication in mammalian hosts is mapped on the structure. PA-497R is not exposed on the molecular surface of this model. The cap binding domain is indicated by purple circles.
